# Hypertrophic non-union of a pathological forearm fracture secondary to multiple myeloma: a case report

**DOI:** 10.1186/1749-799X-5-26

**Published:** 2010-04-21

**Authors:** Tosan Okoro, Robert U Ashford

**Affiliations:** 1 School of Medical Sciences, Bangor University, Bangor LL57 2AS, UK; 2 Department of Orthopaedics, Ysbyty Gwynedd, Bangor LL57 2PW, UK; 3 University Hospitals of Leicester NHS Trust, Leicester LE1 5WW, UK

## Abstract

Skeletal lesions in multiple myeloma are predominantly lytic and when non-union of pathological fractures occur it is typically atrophic. We report a lady of 61 years of age with myeloma who presented with a pathological fracture through an ulnar myeloma deposit. The fracture was immobilised initially then irradiated. Nine months later she re-presented with marked forearm pain particularly on rotation. Radiographs demonstrated a hypertrophic non-union of a pathological fracture with a typical elephant's hoof appearance. The fracture was immobilised using an ulnar nail. Whilst non-unions in metastatic malignancy are typically atrophic, just occasionally hypertrophic non-unions can occur. Management principles remain the same with stabilisation of the entire bone and early mobilisation being appropriate.

## Background

Multiple myeloma (MM) is an incurable disease that is characterised by the accumulation of clonal plasmocytes in the bone marrow [1]. It accounts for 10-15% of all haematological malignancies and 1-2% of all cancers [1]. MM occurs in Europe in approximately 4 out of every 100,000 individuals [2] Approximately 10-40% of patients are asymptomatic at diagnosis [1] whilst 50-70% of MM patients have bone pain due to lytic lesions and pathological vertebral fractures [1].

The characteristic bone lesion seen in myeloma is a sharply defined small lytic area with no reactive bone formation arising in the medulla; the absence of bone sclerosis is due to an inhibition of osteoblastic activity [3]. Involvement of the cortex causes characteristic endosteal scalloping with invasion of the periosteum and occasionally extraosseous extension [4]

Radiotherapy often forms an important part of management and can lead to resolution of bone lesions [1]. We report a case where an undisplaced pathological fracture, treated by a short period in a below elbow cast and subsequently by external beam radiotherapy, went on to form a hypertrophic non-union.

## Case Presentation

**Figure 1:**
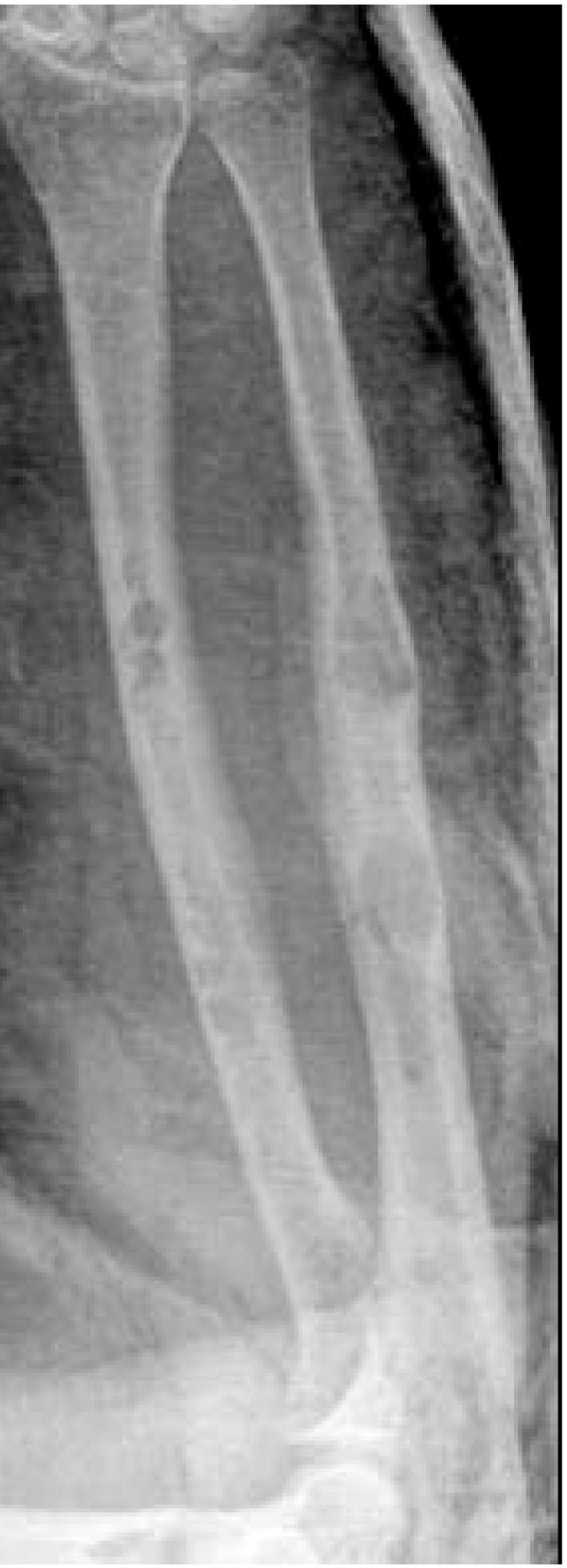
**Pathological fracture through the right ulna with resolution of the born deposit**.

A 61 year old lady with multiple myeloma, diagnosed nine years previously presented to our fracture service with pain in her right forearm. On examination it was painful over the mid-aspect of her forearm with no superficial erythema or swelling. She was neurovascularly intact. Radiographs revealed an undisplaced pathological fracture of the ulna (Figure 1).

Radiographic review of the ulna fracture at 7 weeks showed that there was an attempt at bony union along with an improvement in her symptoms, therefore no further orthopaedic intervention was planned at the time. External beam radiotherapy to this lesion was subsequently arranged by the oncologists.

She was re-referred to the Orthopaedic Oncology service after 9 months with increasing pain in her right forearm whilst performing specific tasks such as cleaning her teeth.

**Figure 2:**
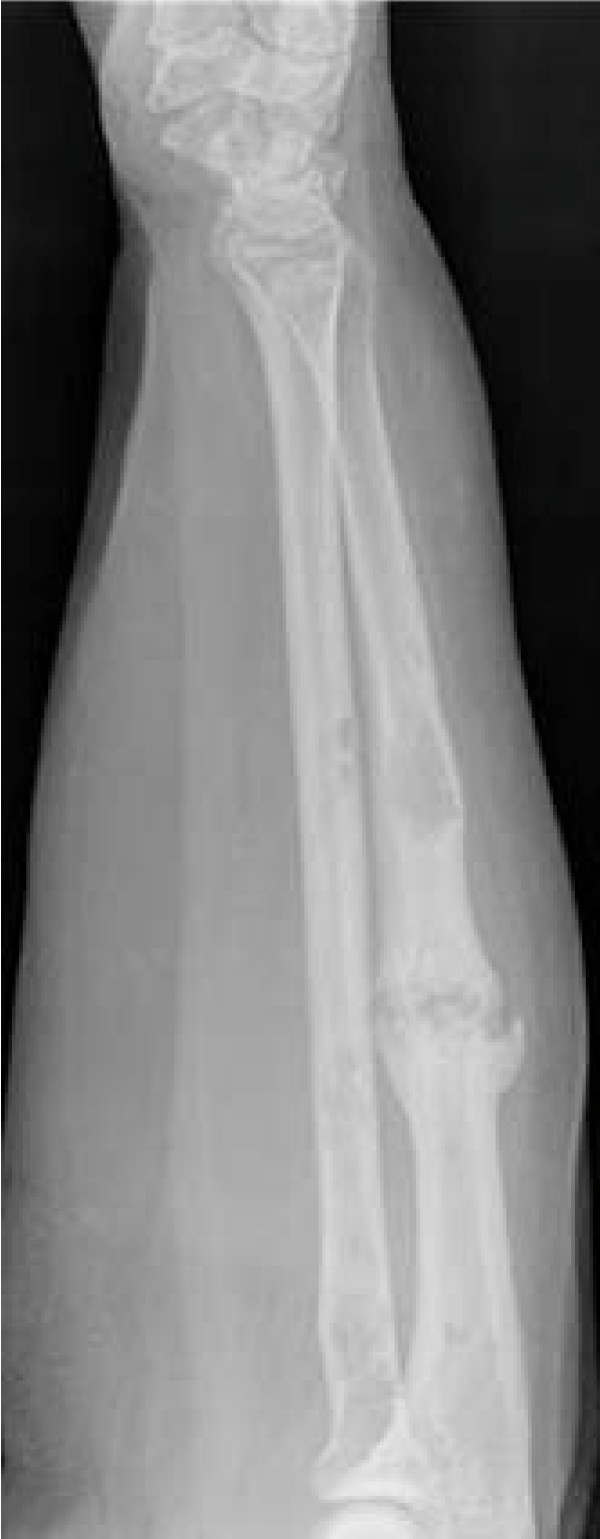
**Hypertrophic non-union of the pathological fracture through the right ulna**.

She had marked pain on pronation and supination of her forearm. Visual Analogue scoring (VAS) of her pain was 9 out of 10. Radiographs demonstrated that she had gone on to develop hypertrophic non-union (with a typical elephant's hoof appearance) of her pathological ulna fracture (Figure 2). The original large myelomatous deposit was no longer evident.

**Figure 3:**
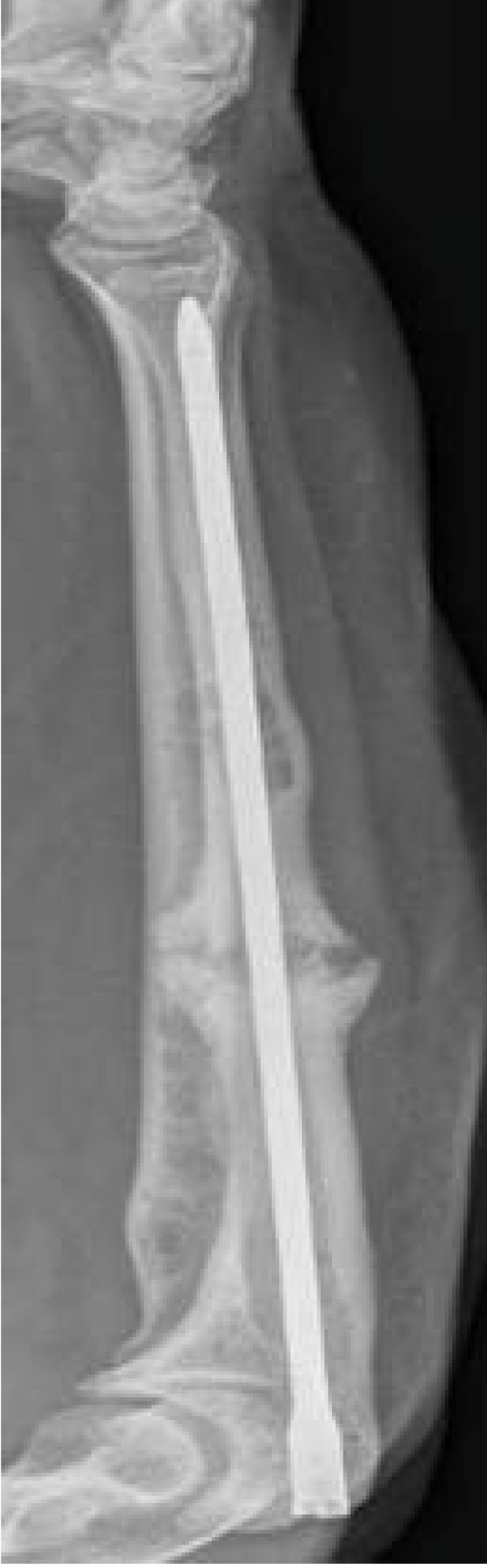
**Anteroposterior X-Ray view of intramedullary fixation of pathological fracture of the right ulna in the immediate postoperative period**.

The fracture was stabilised with a reamed Foresight ulnar nail (Smith and Nephew, Warwick, UK, Figure 3), in an attempt to alleviate her symptoms and achieve bony union.

**Figure 4:**
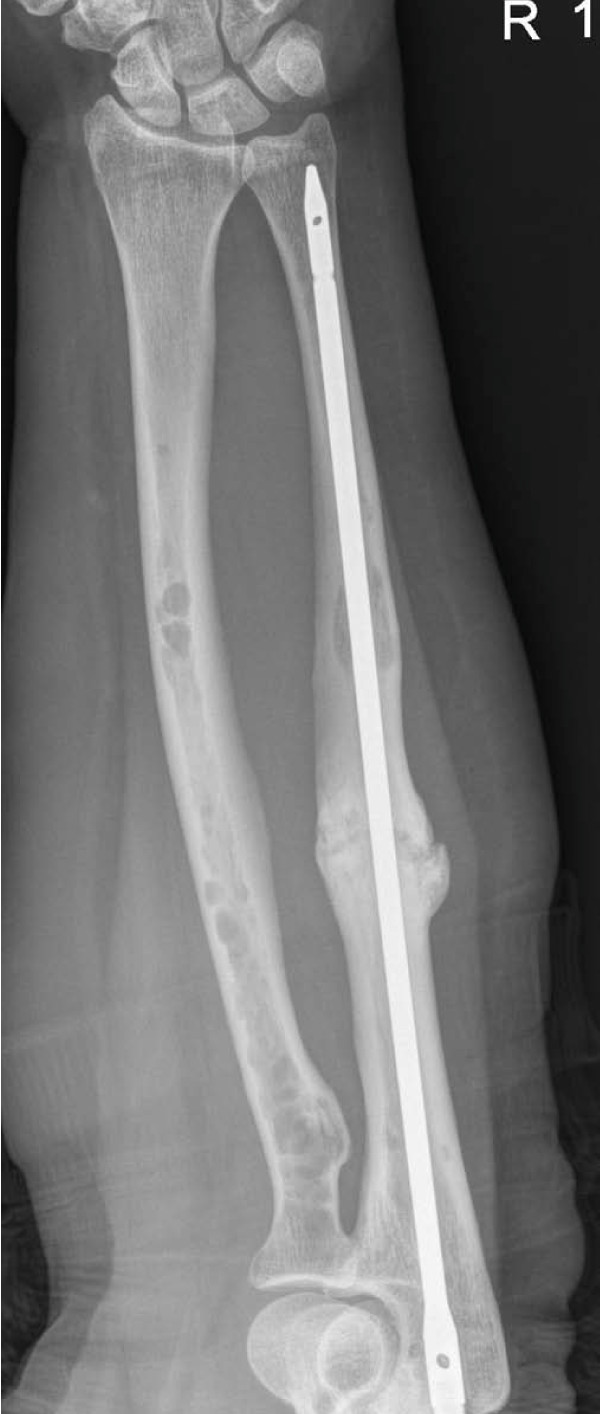
**Radiographic union of pathological fracture of right ulna at 12 months post-operatively**.

On review at 3 months post-operatively, she was symptomatically much improved with pain free forearm rotation. Her VAS had reduced to 2 out of 10. Forearm flexion, pronation and supination were full but extension lacked the last 10°. Radiographically the fracture has united after 12 months of follow up (Figure 4).

## Discussion

Forearm lesions and pathological fractures are relatively rare in multiple myeloma. The commonest sited of fracture are the spine (55%-70% of patients) especially in the lower thoracic or lumbar vertebral bodies [5]. Other common sites of fracture include the femur, pelvis, ribs, and humerus [5]. Fractures result from direct myelomatous involvement of the bone and also can result from the generalized bone loss that is a hallmark of myeloma [6].

Radiation therapy for the treatment of bone tumours and soft-tissue sarcomas may deliver damaging doses of radiation to skeletal bone [7]. It is known that ionizing radiation has a detrimental effect on cortical bone [8] and that it may inhibit and delay fracture union [9].

Failure of bone healing or non-union results from an arrest of the healing process. A non-union that occurs despite the formation of a large volume of callus around the fracture site is commonly referred to as a hypertrophic non-union whilst in an atrophic non-union, little or no callus forms and bone resorption occurs at the fracture site [10]. Fractures through bone involved with malignancy such as myeloma in this instance often will not heal unless the neoplasm is treated [10]. Subperiosteal new bone and fracture callus may form, but the mass of malignant cells impairs or prevents fracture healing, particularly if the malignant cells continue to destroy bone. The radiotherapy administered in this case has once again led to radiographic resolution of the bone lesion [1]. However, this radiotherapy has likely also interrupted the initial attempt at bone union. We postulate that there was an inherent lack of stability in the ulna fracture as upper extremity pathologic fractures are often subjected to distractive forces inherent in lifting and pulling [11]. Although radiotherapy has been shown to help relieve pain in myeloma bone disease with success rates of 50-80% [12], a lack of stability and the radiotherapy would potentially explain the increased hyper vascular response of the callus and the hypertrophic non-union. The use of a reamed nail was in an attempt to achieve union. Most recent radiographs demonstrate satisfactory progression of the lesion towards union (figures 3 and 4). A 9 month interval from radiotherapy to re-presentation indicates that there was an initial satisfactory response to bone pain alleviation but the fracture became the important issue not the tumour. This was the indication for surgical fixation using the intramedullary device.

## Conclusions

Whilst non-unions in metastatic malignancy are typically atrophic, just occasionally hypertrophic non-unions can occur. This is a rare occurrence and the management principles remain the same with stabilisation of the entire bone and early mobilisation being appropriate.

## Consent

Written informed consent was obtained from the patient for publication of this case and any accompanying images. A copy of the written consent is available for review by the Editor-in-Chief of this journal.

## Competing interests

The authors declare that they have no competing interests.

## Authors' contributions

*TO *assessed the patient, participated at surgery and was responsible for drafting the article and collating all relevant images; *RUA *conceived the idea for the case report, performed the surgery and was involved in review of the manuscript. All authors read and approved the final manuscript.
